# Supplementary material for: Does family life education influence attitudes towards sexual and reproductive health matters among unmarried young women in India?
Source: PLoS One. 2021 Jan 25;16(1):e0245883. doi: 10.1371/journal.pone.0245883 (PMC7833152; doi:10.1371/journal.pone.0245883)
Supplement: Attachment — Submitted filename: Response to Reviewer_11102020.docx [file pone.0245883.s001.docx]

**Author’s Response to Reviewers Comment**

**Reviewer #1:** This paper uses nationally representative data to assess exposure to family life education, and the association between exposure to family life education and knowledge about reproductive health issues (e.g., contraception, STIs, and HIV).

I have a few overall concerns about the analysis:

1. Knowledge of reproductive health outcomes was measured with several items. Why were the items treated as separate outcomes, instead of added for singles scores (i.e., one score on knowledge/awareness of contraception; one score on knowledge/awareness of HIV; one score on knowledge/awareness of STIs)? There is likely correlation among items in each set of questions.

Reply: Thank you for a very interesting and valuable suggestion. We very much appreciate it, and accordingly generated five different indices based of several relevant questions to measure the pertinent dimensions related to sexual and reproductive health matters, including index of menstruation, index of reproduction, index of contraception, index of RTI/STI, and index of HIV/AIDS. We have used these five indices as outcome variables in the multivariate analysis to examine the net effect of access to family life education, after accounting for the confounding demographic and socioeconomic factors.

2. It is unclear why family life education would be associated with menstrual problems (it is, but a justification for thinking that such education is related to such problems needs to be provided).

Reply: Previous studies have shown that menstrual problem, among others, is related to knowledge, skill and management of healthy menstrual hygiene. Issues of fear, embarrassment, social taboos, ignorance, and other social barriers negatively impact menstrual health among young women. On the other hand, use of appropriate and safe materials including sanitary/cotton pads, antiseptic liquid, use of clean/dry cloth, avoiding old cloths etc. might help to control menstruation related problems. Therefore, proper information, skill, and education related to SRH is considered to be positively associated with better physical development and management of healthy menstrual hygiene, thus reducing the likelihood of menstruation related problems (Kabir, Saha and Gazi, 2015; Chandra-Mouli and Patel, 2017; Achora et. al., 2018). However, we have modified our dependent variable, as discussed in Point no. 1, that now strictly captures issues related to the management of menstrual hygiene.

3. It is unclear if weights were used for the analysis. If not, why not?

Reply: Survey weights has been used in the data analysis. The same has been reported in the Data Analysis section.

Other comments

1. The abstract mentions two data sets, but the results are based on only one data set.

Reply: The main analysis of the present study is entirely focussed on DHLS-3 (2007-2008) dataset. Although, sub-national data from the Youth in India Study (2006) has also been used to provide corroborative evidence on the role of FLE towards improving the SRH among young men and women in selected states of India. However, we are now dropping the same to avoid any confusion as it does not alter/modify the finding of our study.

2. The introduction/background section can be streamlined, so as to focus on the importance of family life education to knowledge and reproductive health behaviors. I understand that improved reproductive health outcomes for young women may lead to other development outcomes, but that is beyond the scope of the analysis/paper.

Reply: Thanks for the comment. We agree to your kind observation and have incorporated it in the manuscript.

3. The reproductive health knowledge variables need to be described in the text (it wasn't until I read the tables that I understood the analysis was looking at knowledge/awareness and not reproductive behaviours).

Reply: Thanks for your comment. We have provided a brief description of the outcome variables in the Section 2.1 under the Data and Methods section. We have also provided a summary table that provides a detailed description of all the variables used for computing the five indices to measure different dimension of sexual and reproductive health related matters among young unmarried women in the study (Table 1).

4. It is not clear, in table 1, whether any of the variables are significantly associated with exposure to family life education.

Reply: Thanks for the comments. The same has been incorporated.

**Reviewer #2:** Thank you for the invitation to review this insightful paper. Though its of interest, there are essential issues that need to be addressed for it to be publishable.

General comments:

Authors should format the paper to the journal’s requirements. For instance, “Context” as used in the abstract section should be changed to “Introduction”.

A thorough proofreading is required to rectify the typo and grammatical issues. There are several occasions where “adolescent” is used instead of “adolescents”, first sentence of third paragraph of introduction should bear “depict” not “depicts”, “unpleasant state” “These includes” and several others.

Reply: Thanks for your comment. We have replaced the “Context” with “Introduction” as suggested by the reviewer. The manuscript has been edited by a professional English editor to improve the overall flow of the paper and correction of grammatical/typographical errors.

Abstract

1. Under Context: revise to “…adolescents and young people…” not “…adolescent and young people…”

Reply: Thanks for the comment. It has been incorporated.

2. The author should remove “etc”

Reply: Thanks for the comment. It has been incorporated.

Introduction

3. Second paragraph: The author wrote “At many international negotiable platforms, including Programme of Action of the International Conference on Population and Development (ICPD, 1994), the World Plan of Action for Youth of the United Nations General Assembly (1995) and ICPD+15 2009, the global agenda was streamlined for empowering the situation of adolescents and youth [13–15]”. The conferences aimed at empowering the youth but not “empowering the situation of the youth” as indicated. This should be rectified.

Reply: Thanks for the comment. It has been incorporated.

4. This sentence should be referenced: “Majority of these youth reside in the poorest and least developed countries of Asia, Africa and Latin America which together constitute over 85 percent of world’s youth population.”

Reply: Thanks for the comment. In order to improve the focus on SRH issues among adolescent and youth population, we have dropped couple of paragraphs that highlighted broader socioeconomic and developmental issues connected to young people. The above lines have also been dropped.

5. The author has done well by acknowledging some initiatives/policies in the country. Is there any data/evidence on how these have helped improved the attitude towards sexual and reproductive health?

Reply: Thanks for the comment. As suggested by the reviewer, we have added the evidence to highlight the role of some of the important policies and programmes on the health and well-being of adolescent and youth population.

6. The author wrote “India is one of the youngest nations…” what is the basis for this or is the author implying that it has a high proportion of young women/men globally?

Reply: Thanks for the comment. As rightly underlined by the reviewer, this sentence laid emphasis upon the relatively higher proportion of young population in India relative to other countries in the world.

7. The background needs to be summarised, it is too bulky. The historical accounts can be truncated and rather project the Indian context.

Reply: Thanks for the comment. As suggested, we have tried to reduce the background section wherever possible and focus on the SRH issues among adolescent and youth, particularly in the Indian context.

Methods

8. One of the outcome variables is “menstruation related problems”. Most of the problems are biological and conceptually does not make sense to investigate with Family Life Education.

Reply: Thanks for the comment. As suggested by the reviewer, we have modified the outcome variable. We generated an index of menstruation with focus on the questions that highlights the management of menstrual hygiene in particular such as experience of any menstruation related problem in last three months; methods for protection during menstrual cycle such as cloth, locally prepared napkins, sanitary napkins.

9. How was “family life education” as an outcome variable measured and what does it constitutes? Explanation is required here.

Reply: Thanks for your comment. It is briefly discussed in the Methods section. The DLHS-3 asked unmarried women 15-24 years respondents - “*Are you aware of family life education/sex education programme, i.e., about bodies, growing up, male-female relationship, and sexual matter?*” Subsequently, the young women respondents were asked- “*Have you ever received any family life/sex education?*” and further study participants were asked- “*If yes, where did you receive that?*” The second question mentioned above was used to generate the binary variable of family life education (FLE).

10. Change “Methods” to “data analysis”

Reply: Thanks for the comment. It has been incorporated as suggested.

Results

11. The author can present about three regression results per table to make it more presentable.

Reply: Thanks for your comment. We have modified the regression analysis as suggested by the reviewer. There are two tables of regression analyses. First table presents the empirical results from the binary logistic regression model. Second table presents the result from set of multiple linear regression model.

Discussion and conclusion

The author spends more time describing other studies in some instances instead of situating the present study within the existing literature.

For instance, this occupies a full paragraph but not informative enough for a discussion “Another household level representative survey entitled ‘Youth in India: Situation and Needs’ was conducted in 2006-07 across six Indian states [26]. The main objective of this survey was to gather evidence on key life transitions experienced by youth as well as their awareness, attitudes and life choices. The study was conducted in the following selected Indian states: Andhra Pradesh, Bihar, Jharkhand, Maharashtra, Rajasthan and Tamil Nadu. Overall, about 50,773married and unmarried young women and men were successfully interviewed, from 1,74,037 sample households. Unmarried men and women as well as married women (15-24 years) were interviewed, whereas the age group for married men was extended to 15-29 years, in the first ever 22 landmark survey on youth in India. The results from this survey lend support to the findings of our study”

Reply: Thanks for the comment. The above paragraph has been dropped and focus has been made to emphasize upon the findings and implication of the results coming from our study.
