# Supplementary material for: Does family life education influence attitudes towards sexual and reproductive health matters among unmarried young women in India?
Source: PLoS One. 2021 Jan 25;16(1):e0245883. doi: 10.1371/journal.pone.0245883 (PMC7833152; doi:10.1371/journal.pone.0245883)
Supplement: Attachment — Submitted filename: Response to Reviewer_03122020.docx [file pone.0245883.s002.docx]

**Author’s Response to Reviewers Comment**

**Reviewer #1:**

I would not treat the measures of knowledge as individual items in bi-variate analysis.  It isn't clear that the level of detail is warranted.  Also, kindly address the comments of the review below. The paper needs copy editing.

Reply: Thanks a lot for this important comment.

We have accordingly incorporated and clarified the use of separate variables/items related to knowledge and awareness of reproductive health matters in Data and Methods section as follows-

*‘In the bivariate analyses, we separately used individual variables/items related to different measures of knowledge and awareness of reproductive health matters to highlight the variability across the access to family life education among young unmarried women’.*
